# Nonlinearity in Memristors for Neuromorphic Dynamic Systems

**DOI:** 10.1002/smsc.202100049

**Published:** 2021-09-28

**Authors:** Ke Yang, J. Joshua Yang, Ru Huang, Yuchao Yang

**Affiliations:** ^1^ Department of Micro/nanoelectronics Peking University Beijing 100871 China; ^2^ Electrical and Computer Engineering Department University of Southern California Los Angeles CA 90089 USA; ^3^ Center for Brain Inspired Chips Institute for Artificial Intelligence Peking University Beijing 100871 China; ^4^ Center for Brain Inspired Intelligence Chinese Institute for Brain Research (CIBR) Beijing 102206 China

**Keywords:** chaos, coupled oscillatory networks, neuromorphic dynamic systems, nonlinearity in memristors, reservoir computing

## Abstract

As semiconductor technology enters the more than Moore era, there exists an apparent contradiction between the rapidly growing demands for data processing and the visible inefficiency rooted in traditional computing architecture. Neuromorphic systems hold great prospects in enabling a new generation of computing paradigm that can address this issue, which demands device components with rich dynamics and nonlinearity. Herein, the nonlinearity in memristive devices and their application in building neuromorphic dynamic systems are reviewed. The internal mechanisms that endow memristive devices with nonlinearity and rich dynamics are reviewed and subsequently the nonlinear spiking neurons that are implemented utilizing the physical processes in memristors are shown. Typical examples on neuromorphic dynamic systems based on nonlinear memristors are summarized, including memristive reservoir, memristive oscillatory neural network, and memristive chaotic computing. Finally, an outlook in the development of neuromorphic dynamic systems is given.

## Graphical abstract

Herein, the internal nonlinearity and rich dynamics in memristive devices, distinguishing them from static linear circuit elements, which provide vital substrates for building neuromorphic dynamic systems capable of information processing with high efficiency, are discussed. Typical examples of neuromorphic dynamic systems based on nonlinear memristors, including memristive reservoir, memristive oscillatory neural network, and memristive chaotic computing, are highlighted.

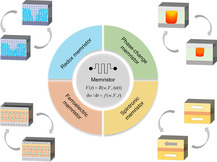

## 1 Introduction

Recent years have witnessed dramatic advances in deep learning technology, especially for image and video recognition, as well as natural language processing.^[^ 1, 2, 3 ^]^ Different from previous scientific computing tasks involving relatively small amount of data, the heavy vector matrix multiplications (VMMs) involved in the artificial neural network (ANN) algorithms require huge data movement in traditional von Neumann hardware, which is featured by separated computing and memory units and therefore places an inevitable limitation on the computational throughput and energy efficiency. To address this issue, a direct technical route is to blur the boundary between computing and memory and conduct in situ VMM operations in the memory array. Computing‐in‐memory (CIM) architectures based on emerging resistive switching devices, which fall into the general category of memristors, including resistive random access memory (RRAM), phase‐change memory (PCM), magnetic random access memory (MRAM), etc.,^[^ 4, 5 ^]^ have become promising candidates for the CIM technology due to their non‐volatile characteristics, analog memory states, and high integration density. In general, the matrix elements are mapped to the conductance of the corresponding devices in the memristor array, whereas the vector is converted into the input voltages on the rows. Under this configuration, the result of VMM operation can be obtained physically according to Ohm's law and Kirchhoff's current law in one read cycle and extracted by column currents. Such principles for VMM operation have been successfully demonstrated on the acceleration of perceptron,^[^ 6, 7, 8 ^]^ convolutional neural network (CNN),^[^ 9, 10 ^]^ long short‐term memory (LSTM),^[^ ^11^ ^]^ Hopfield network,^[^ ^12^ ^]^ and so on. Similarly, VMM on memristor array has also facilitated the hardware implementation of other machine learning algorithms, including K‐means clustering^[^ ^13^ ^]^ and sparse coding,^[^ ^14^ ^]^ as well as scientific computing tasks such as the solution of partial differential equation^[^ ^15^ ^]^ and matrix equation.^[^ ^16^ ^]^ These recent explorations have demonstrated the potential of memristors in the construction of accelerators, by taking advantage of the efficient VMM operations directly utilizing physical laws for linear physical computing. In addition to these deterministic operations, intrinsic stochasticity involved in memristive devices can also be utilized for security applications. Physical unclonable function and hardware fingerprint as hardware identities can be implemented with device variations; meanwhile, memristive random number generators offer a low‐cost solution for key generation.^[^ ^4^ ^]^

**Figure 1:**
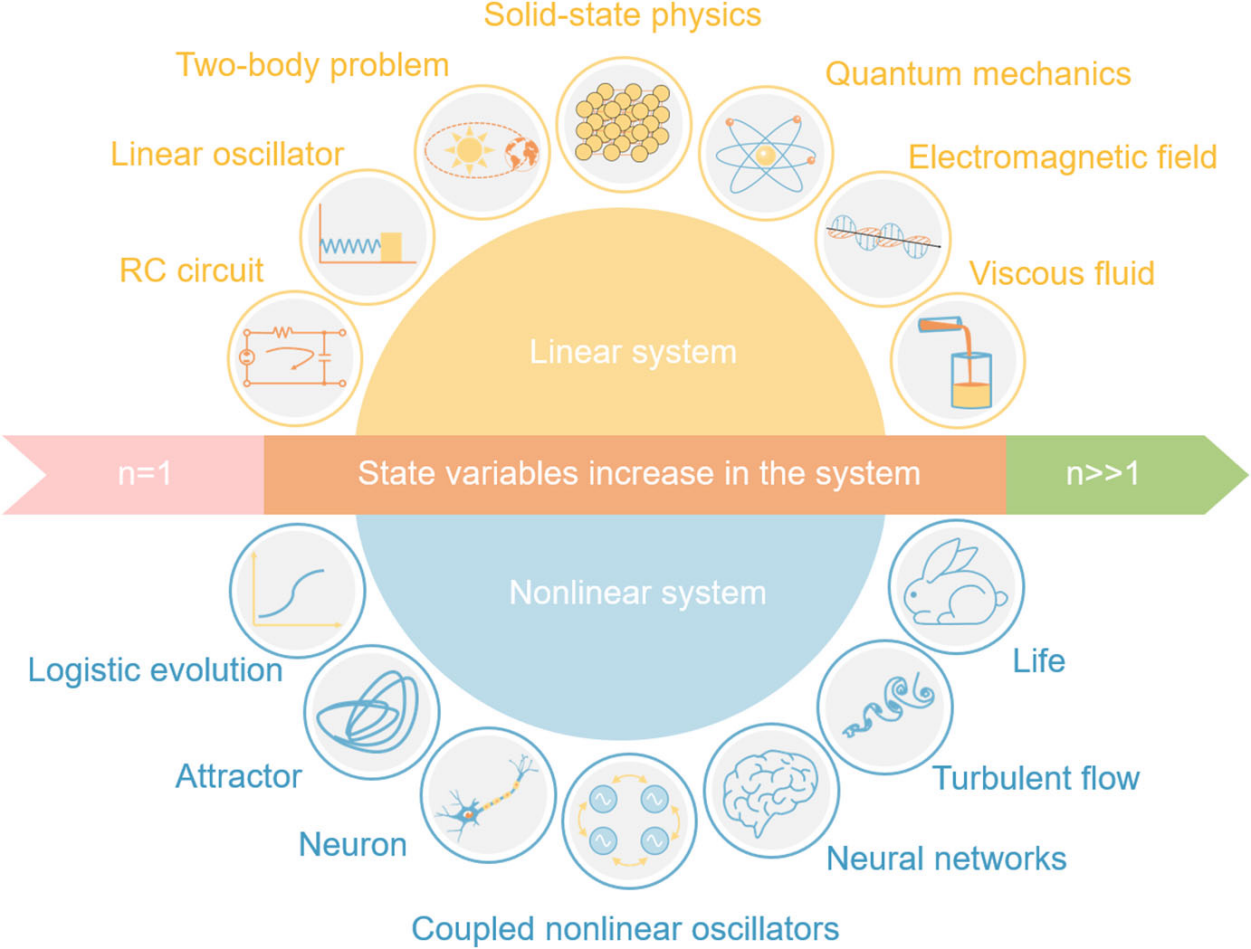
Illustration of linear and nonlinear systems in different dimensions. Examples of linear systems are illustrated in the upper part, whereas the nonlinear ones are in the lower part. The dimensionality (order) of the system, which determines the complexity and dynamic property of the system, increases from left to right.

However, ANNs usually suffer from much higher power consumption compared with biological neural networks, and the tasks that can be conducted by ANN remain limited, in contrast to the general intelligence, advanced cognitive functions, as well as thinking and learning abilities of the human brain. This may lie in the fact that ANN, as a highly simplified and abstract model of the nervous system, has lost many of the key dynamics of the brain. The exploration of neuromorphic systems in real sense will be based on the understanding of high‐dimensional nonlinear systems, which have been much less understood compared with the linear systems. As shown in **Figure** **1**, the simplest systems that we are familiar with are usually low‐dimensional linear systems carrying simple dynamic properties, including growth, decay, as well as equilibrium in a 1D resistor‐capacitor (RC) system and oscillation in a 2D mass and spring system. High‐dimensional linear systems can normally be broken down according to the superposition principle and treated by linear analysis methods, like Fourier analysis. However, when entering the nonlinear regime, rich dynamics will emerge, and the complexity increases as the dimension increases in the system. One will get fixed points and bifurcations in 1D nonlinear systems, nonlinear oscillation in 2D nonlinear systems, whereas chaos and fractals when dimension is higher than three.^[^ ^17^ ^]^ Interestingly but not surprisingly, the brain is constructed as a high‐dimensional nonlinear system. For instance, the neuron is proven to operate at a state that is highly active while maintaining its stability called edge of chaos,^[^ 18, 19 ^]^ and the brain is composed of 10^11^ such neurons coupled with each other to generate complex dynamic patterns, which may be the key to the emergence of real intelligence. The limitations existing in present ANN accelerators and the computational potentials of nonlinear dynamics provide strong motivations for us to build a brain‐inspired system in a nonlinear and dynamic manner.

**Figure 2:**
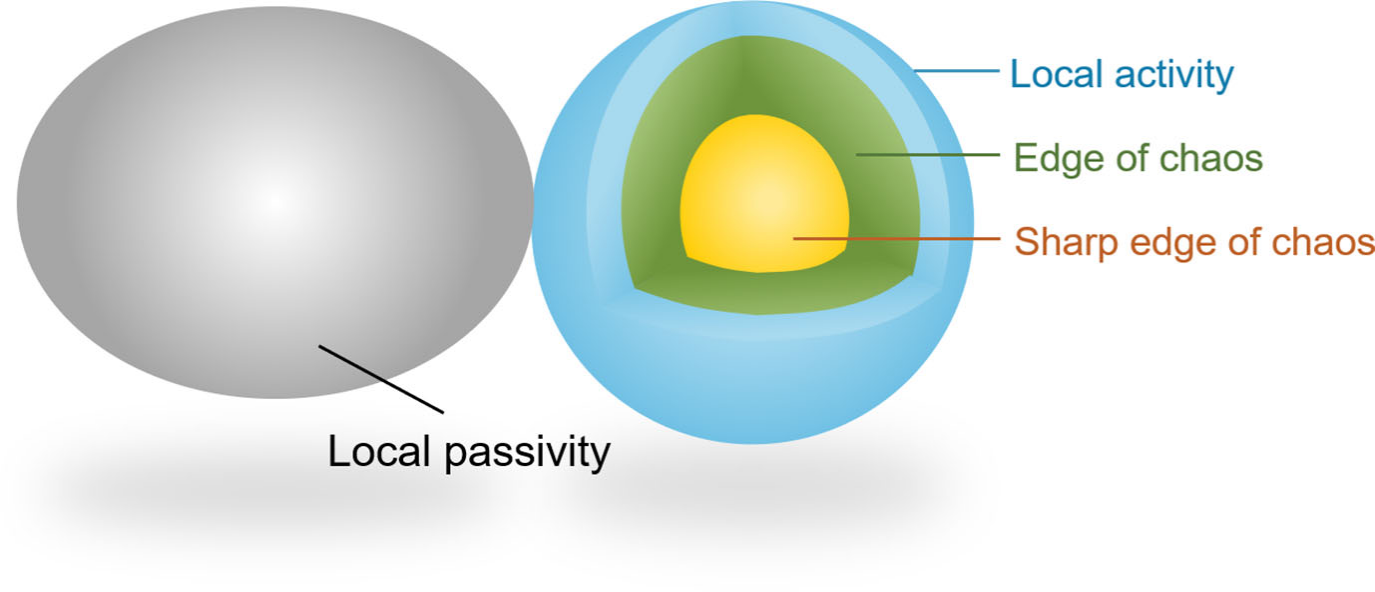
Illustration of Chua's theory of local activity.

As shown in **Figure** **2**, the neural activity only emerges within the regime of edge of chaos. Equipped with such local activity, neurons as information processing units in the nervous system exhibit various spiking behaviors, enabling efficient spatiotemporal integration^[^ ^20^ ^]^ and nonlinear computation functions.^[^ ^21^ ^]^ Local activity is a necessary condition for complexity emergence in the system. The complexity here describes that a spatially continuous or discrete medium made of identical cells interacting with all cells located within a neighborhood can generate nonhomogeneous static or spatiotemporal patterns under homogeneous initial and boundary conditions.^[^ ^22^ ^]^ Otherwise, all solutions of the nonlinear system will converge to some specific steady state when time goes to infinity. The definition of local activity required that the energy of the small signal absorbed by the device in a period is negative, which is mathematically described as Δ*e*(*t*)  =  $\int_{0}^{t}$ Δν(*s*) ×  Δ*i*(*s*) ×  *ds* <0, where Δν is an infinitesimal voltage perturbation at the certain operating point of memristor.^[^ ^23^ ^]^ Another familiar example of the typical locally active device is the transistor, which converts a small input signal into a large output signal with extra energy supply. Usually, the locally active operating point of a memristor can be found by using local activity theorem.^[^ ^22^ ^]^ Recent experimental results show that such local activity can be contained in memristors,^[^ 18, 20, 23, 24 ^]^ which will be introduced in the following section. Such nonlinear dynamics is always regarded as nonideal characteristics when the memristor serves as reconfigurable resistive memory for VMM acceleration. These nonlinearities are possibly derived from the nonlinear physical processes involved during resistive switching including phase transition between crystallization and amorphous state, the ferroelectric polarization flip, spin–torque‐induced magnetization switching, and magnetic domain wall motion.^[^ ^4^ ^]^ It may also come from the internal nonlinear chemical reaction in the switching process. Likewise, nonlinearities may also stem from the complex physical–chemical coupled process such as ion drift, which involves redox or composition change to keep charge neutrality.^[^ 25, 26, 27, 28 ^]^ From a bionic point of view, the technologically relevant memristors at present that are based on ion transport^[^ 25, 26, 27, 28 ^]^ are similar to the working modes of synapses and neurons in the nervous system, thus offering an opportunity to emulate the biological components faithfully beyond behavioral level. More importantly, rich nonlinear dynamics is naturally embedded in the ion transport involved and the subsequent filament formation/rupture process, which provides a physical substrate for the implementation of more complex nonlinear operations and construction of intelligent neuromorphic systems.

In this article, we review the nonlinearity in memristive devices and its application in building neuromorphic dynamic systems. We first show the mechanisms behind the memristor nonlinearity and subsequently discuss the nonlinear spiking neurons that are implemented utilizing the physical and chemical processes in memristors. Typical examples on neuromorphic dynamic systems based on nonlinear memristors are summarized, including memristive reservoir, memristive oscillatory neural network, and memristive chaotic computing. Finally, we give an outlook on the development of neuromorphic dynamic systems.

## 2 Mechanism behind Memristor Nonlinearity

Memristor, the abbreviation of “memory resistor,” is originally defined to describe the relationship between electric charge *q* and magnetic flux *φ*, that is, dφ=Mdq.^[^ 29, 30 ^]^ As the electric charge and magnetic flux are the integration of current *i* and voltage *v* with respect to time, respectively, *M* has the same dimension as the resistance and is called memristance. In stark contrast to the resistor in the traditional sense, a memristor has the ability to retain the memory of the external electrical stimulations in its resistance.^[^ ^31^ ^]^ In contrast to the clear and concise definition of theoretical memristors, it remains controversial whether experimentally realized resistive‐switching memory cells are memristors or not.^[^ 32, 33 ^]^

This concept of memristors can be further generalized to^[^ ^34^ ^]^

(1) v=M(w,i)i

(2) $$\frac{dw}{dt}=g(w,i)$$

where the memristance is determined by the input current *i* and an internal state variable *w.* Similarly, a voltage‐controlled memristor is mathematically described as

(3) i=G(w,v)v

(4) $$\frac{dw}{dt}=f(w,v)$$

in which the device conductance is not only decided by the present input but also reflects the historical influences by voltage stimuli on its state.

Unlike a normal resistor whose value is either fixed or determined by the instantaneous input, here, for a memristor, its state is determined by one or a set of internal state variable(s) *w*. The dynamic effect or the change rate of the state variable *w* is related to the instantaneous inputs, and hence the value of *w* and the device state can only be obtained from a time integral, which fundamentally contributes to the history‐dependent behaviors and intrinsic nonlinearity of memristors.^[^ ^35^ ^]^ By identifying the conduction mechanism (*i–v*), state variable(s), and dynamic equations to the actual physical processes in specific memristive devices, different devices can be modeled within the aforementioned memristor theoretical framework.

The physical embodiments of the memristors correspond to various resistive switching devices, which can be categorized into four classes according to their switching mechanism: redox reaction, phase change, magnetic polarization, and ferroelectric polarization.^[^ ^4^ ^]^ These mechanisms endow the memristors with rich nonlinear dynamics and thus provide us opportunities for constructing biologically plausible dynamic computing systems.

### 2.1 Redox‐Based Memristors

In a redox‐based memristor, the resistive switching stems from redox reactions and ion transportation under electric field, chemical potential gradient, and temperature gradient.^[^ 36, 37, 38 ^]^ Depending on the dominant ions involved in the switching process, the redox memristors are further divided into valence change memory (VCM) and conductive bridge random access memory (CBRAM).

**Figure 3:**
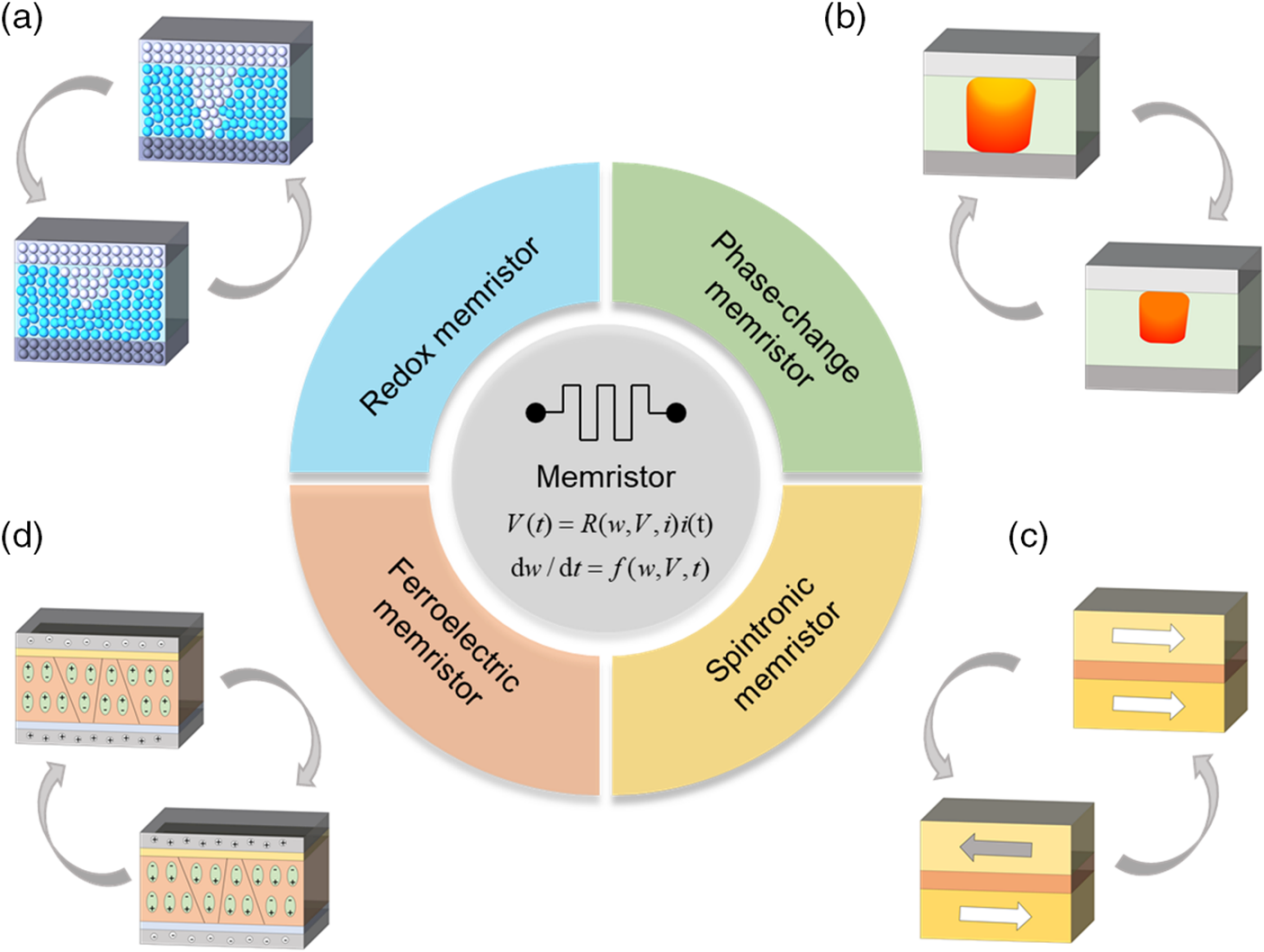
Memristors with different mechanisms. a) Illustration of a redox‐based memristor. b) Illustration of a phase‐change memristor. c) Illustration of a spintronic memristor. d) Illustration of a ferroelectric memristor.

VCM is usually based on transition metal oxides and perovskites, where anions, like oxygen ions (oxygen vacancies), show a relatively high mobility under electric field and thermal effect. When stimulated by a voltage across the device, a conducting channel can be formed between the electrodes via the migration and accumulation of oxygen vacancies, and the resultant filaments are usually composed of a suboxide phase, leading to a high conductance state,^[^ 39, 40, 41 ^]^ as shown in **Figure** **3**a. Taking TaO_*x*_‐based VCM device as an example, redox resistive switching device can be formulated as a generalized memristor.^[^ ^42^ ^]^ Here, the history‐related state variable can be abstracted as the volume fraction of the metallic filament and denoted as *y*.^[^ ^42^ ^]^ The filament is considered to have a linear current–voltage dependence while the remaining 1−*y* part can be described by the nonlinear Frenkel–Poole transport mechanism. Thus, the total current contributed by these two portions is given by

(5) $$i=v[yG_{m}+(1-y)a\text{exp}\;(b\sqrt{\left|v\right|})]$$

with constant *a*, *b*, *G*_m_. In addition, the switching dynamics can be characterized by

(6) $$\frac{dy}{dt}=A\sinh[\frac{v}{\sigma_{\text{OFF}}}]\text{exp}{\left(-\frac{y_{\text{OFF}}}{y}\right)}^{2}\text{exp}[\frac{1}{1+\beta p}](v<0)$$

(7) $$\frac{dy}{dt}=B\sinh[\frac{v}{\sigma_{\text{ON}}}]\text{exp}{\left(-\frac{y}{y_{\text{ON}}}\right)}^{2}\text{exp}[\frac{p}{\sigma_{p}}](v>0)$$

where *A*, *B*, $\sigma_{\text{OFF}}$, $y_{\text{OFF}}$, *β*, $\sigma_{\text{ON}}$, $y_{\text{ON}}$, $\sigma_{p}$ are held constant. Together, the TaO_*x*_ device can be described by the Chua memristor equation i=G(y,v)v and $\frac{dy}{dt}=f(y,v)$.

There are also nonfilamentary memristors based on anion transport in addition to the filament‐type introduced earlier. In some nonfilamentary devices, the redox reaction induced by anion migration occurs at the interface of the two layers of materials and results in a change in the thickness of the interfacial layer, which corresponds to different resistance states.^[^ 43, 44 ^]^ This nonfilamentary resistive switching may also originate from the modification of the Schottky barrier caused by the accumulation of anions at the interface.^[^ 45, 46, 47 ^]^ Such a nonfilamentary device avoids the randomness caused by the formation and fracture of the filament and thus is expected to have better device uniformity and lower variation.^[^ ^48^ ^]^

A form of nonlinearity in VCM devices is the nonlinear conductance modulation in response to identical applied pulses. Note that the nonlinearity here does not refer to the nonlinearity of the current–voltage relation at each resistance state. Several theories have been proposed to explain this nonlinear phenomenon.^[^ 40, 41, 49, 50, 51 ^]^ In VCM devices, where the resistive switching is dominated by localized, filament region(s), the SET and RESET processes are realized by the formation and dissolution of oxygen‐deficient filaments based on oxygen ions/vacancies transport. In the initial stage of SET (RESET), the formation and dissolution of the filament involves the ions/vacancies which are adjacent to the filament, and the movement is accelerated by the higher temperature around the filament region, hence resulting in abrupt increase (decrease) in the conductance. However in the subsequent SET (RESET) stage, the ions/vacancies need to travel for longer distance and ion migration is retarded by the temperature profile, which is responsible for the more gradual change in the conductance.^[^ ^49^ ^]^ In addition, studies on the impact of filament length indicate that the rapid conductance increase in the initial stage is attributed to the first completeness of the filament to form an integral conductive channel where the electron transport mechanism may be most sensitive to the addition or removal of atoms to the conduction path, whereas the subsequent slow increase in conductance is caused by further increase in the filament diameter when the sensitivity of each atom decreases.^[^ ^50^ ^]^

Compared with VCM cells, CBRAM that usually adopts a sandwich structure of electrochemically active electrode/solid electrolyte/inert electrode even exhibits stronger nonlinearity, due to the more abrupt nature of metal filament formation. In general, the metal atoms are oxidized and enter the solid electrolyte when a positive voltage is applied on the active electrode, and a metal filament is gradually formed through redox reactions to bridge the electrodes, which results in an abrupt conductance increase.^[^ 25, 26, 36, 40, 51, 52 ^]^ It should be noted that if the wetting contact angle is large between the active metal and the dielectric in the material system, the filament can break and form a series of nanoparticles through diffusion after the voltage is removed, driven by the minimization of the interfacial energy between the metal filament and the dielectric, offering a source of desirable temporal dynamics.^[^ ^53^ ^]^

### 2.2 Phase‐Change Memristor

A PCM cell consists of a phase‐change layer sandwiched between two metallic electrodes. In general, the phase‐change layer exhibits a high conductance in its crystalline phase while having low conductance in its amorphous phase. The resistive switching of such a device is induced by a reversible transition between amorphous and crystalline phases. To increase the device conductance, a current pulse is applied to heat up the device to a temperature that is higher than the crystallization temperature but lower than the melting point, thus resulting in recrystallization of the amorphous region.^[^ 54, 55, 56, 57 ^]^ However, in the depression process, a current pulse with a sufficiently high amplitude increases the temperature above the melting point, melting a significant part of the phase‐change material. Followed by sudden interruption of the current, the molten material quenches into the amorphous phase due to glass transition.

Another class of the phase‐change device in a broad sense is Mott memristor, which is shown in Figure 3b, and typical material systems include VO_*x*_ and NbO_*x*_.^[^ 58, 59, 60 ^]^ The Mott memristor such as NbO_*x*_ device can be regarded as an extended memristor by specifying temperature *T* as its internal variable,^[^ 24, 61 ^]^ which is expressed as

(8) i=G(T,v)v

(9) $$\frac{dT}{dt}=f(v,T)=\frac{G{\left(v,T\right)}^{2}}{C_{\text{th}}}-\frac{T-T_{\text{amb}}}{R_{\text{th}}\left(T\right)C_{\text{th}}}$$

Here, Equation (8) characterizes the *I–V* relationship with a highly nonlinear transport relationship with internal temperature.^[^ ^62^ ^]^ Meanwhile, the temperature change follows Newton's law of cooling with ambient temperature *T*_amb_, thermal capacitance *C*_th_, and a temperature‐related effective thermal resistance *R*_th_ that is different for the insulating and metallic state.

### 2.3 Spintronic Memristor

In general, a magnetic tunneling junction (MTJ) contains a nonmagnetic spacer sandwiched between two ferromagnetic layers including a free layer and a pinned layer.^[^ 63, 64 ^]^ The magnetization state of the device can be either altered by a magnetic field or spin–torque excitation. When the electric current flows through these junctions, it becomes spin polarized and generates a torque on magnetization, which subsequently induces a sustained magnetization precession at a frequency from hundreds of megahertz to tens of gigahertz. In case of perpendicular anisotropy thin‐film MTJ shown in Figure 3c, the angle *θ* between the free‐layer magnetization and pinned‐layer magnetization can be modeled mathematically by the Landau–Lifshiltz–Gilbert equation as^[^ ^65^ ^]^

(10) $$\frac{d\theta }{dt}=g(i,\theta )=\alpha \gamma H_{k}(-\sin\theta \cos\theta +\frac{\eta \hbar i}{2\alpha eM_{S}H_{k}V}\sin\theta )$$

and the *i–v* relationship of such spintronic memristors can be written as

(11) $$v=R(i,\theta )i=\frac{1}{G_{0}\left(1+\frac{TMR}{TMR+2}\cos\theta \right)}$$

where *G*_0_ is the conductance when magnetization direction of the free layer is perpendicular to that of the pinned layer, and TMR is the ratio of difference between high and low conductance to low conductance.

Magnetization oscillation corresponds to the oscillation in magnetoresistance that can be measured through the voltage. The resultant amplitude of oscillation has a highly nonlinear relationship with respect to the input current and depends intrinsically on previous inputs. This nonlinear oscillator with short‐term memory can be exploited as an oscillatory neuron for reservoir computing and coupled oscillatory neural networks.^[^ 66, 67, 68 ^]^

### 2.4 Ferroelectric Memristor

Ferroelectric materials can maintain reversible remnant polarization, which can be changed by an external electric field.^[^ ^69^ ^]^ In the case of ferroelectric tunnel junction (FTJ), the resistance is decided by the tunneling current, which in turn can be controlled by the polarization state, as shown in Figure 3d. Externally applied positive (negative) voltage pulses that lead to the nucleation or propagation of the down‐polarized (up‐polarized) domains will change the configuration during polarization reversal and result in different resistance states. Such ferroelectric memristors can be described by the following model^[^ ^70^ ^]^ with nucleation time *τ*_N_ and characteristic propagation time *τ*_p_ when the relative fraction of the downward domain *s* serves as the state variable.

(12) v(t)=R(s,v)i(t)

(13) $$\frac{ds}{dt}=f(s,v)=(1-s)\{\frac{2}{\tau_{P}\left(v\right)}(\frac{t-\tau_{N}\left(v\right)}{\tau_{P}\left(v\right)})\}$$

The ferroelectric material can be incorporated into a field‐effect transistor (FET) by serving as the gate dielectric layer sandwiched between the gate electrode and source–drain conduction region of the channel, which forms the so‐called ferroelectric FET (FeFET).^[^ ^71^ ^]^ The remnant polarization in this ferroelectric layer memorizes the past electric stimuli and maintains the conductance of the transistor channel when the gate bias is absent, therefore showing nonvolatile memory. The combination of the gradual polarization of the ferroelectric layer and the threshold characteristics of the FET makes the conductance of the device exhibit a very strong nonlinear response with respect to the number of voltage pulses applied to the gate.

In addition to the memristors based on the above four mechanisms, which are studied as promising emerging memory technology, there are also electrogated memristors and purely electronic‐based devices belonging to this big family. In an electrolyte‐gated device, the ions inside the electrolyte can be driven by the gate electrode and change the channel conductance through electrochemical doping and/or ion intercalation effect.^[^ 72, 73 ^]^ One typical purely electronic switching mechanism is trapping and detrapping. For example, a charge‐trapped transistor can switch the conductance of its channel utilizing the traps in the dielectric layer.^[^ 74, 75 ^]^ Compared with the devices based on ion transport or phase‐transition mechanism, pure electronic‐based devices are expected to be more stable and have faster speed.^[^ ^46^ ^]^ The basic characteristics of the device are of great importance for ensuring the low power consumption, scalability, and reliability of the memristive neuromorphic system. A comparison of the device characteristics of different mechanisms, including device dimension, operating voltage, power consumption, transition speed, ON/OFF ratio, endurance, and retention, is shown in **Table** **1**. A comparison of these basic performances and further explanations can be also found in previous reviews.^[^ ^4^ ^]^

**Table 1:** A comparison between different memristors

Type	State variables	Operating voltage	Power consumption	Speed	Endurance	On/off	Size
Redox	1–2^[^ 40, 179 ^]^	0.3 V^[^ ^180^ ^]^	115 fJ^[^ ^181^ ^]^	85 ps^[^ ^182^ ^]^	>10^12^^[^ ^183^ ^]^	10^6^^[^ ^184^ ^]^	2 nm^[^ ^185^ ^]^
Phase change	1–3^[^ 20, 24, 61 ^]^	0.9 V^[^ ^186^ ^]^	≈1 pJ^[^ ^145^ ^]^	300 ps^[^ ^187^ ^]^	>10^12^^[^ ^188^ ^]^	10^3^^[^ ^189^ ^]^	5 nm^[^ ^190^ ^]^
Spintronic	1^[^ ^65^ ^]^	<1 V^[^ ^191^ ^]^	≈10 fJ^[^ ^192^ ^]^	200 ps^[^ ^193^ ^]^	10^15^^[^ ^194^ ^]^	≈10^[^ ^195^ ^]^	10 nm^[^ ^196^ ^]^
Ferroelectric	1^[^ ^70^ ^]^	<1 V^[^ ^197^ ^]^	100 fJ^[^ ^198^ ^]^	10 ns^[^ ^199^ ^]^	>10^6^^[^ ^200^ ^]^	>10^2^^[^ ^201^ ^]^	20 nm^[^ ^179^ ^]^

Besides the earlier first‐order memristors which have been summarized according to their switching mechanism, a high‐order memristor should be modeled by multiple internal variables and differential state equations. These high‐order memristive systems with coupled state variables can exhibit more complex behavior that originated from its internal dynamics and hence have the potential to emulate some of the crucial processes in biological neuromorphic systems. Here is an example of synaptic plasticity in biology. The efficiency of the synapse is determined by the surrounding concentration of calcium ions, which is influenced by the spike stimuli. This second‐order system can be implemented by a second‐order memristor which contains temperature as the second state variable.^[^ 76, 77 ^]^ A three‐order memristor can generate chaotic oscillation and advanced biological activity, which has also been experimentally demonstrated recently. Related details can be found in the latest section. In addition to the integer‐order memristive element, a memfractive system is also an important supplementary of the memristor theory.^[^ 78, 79 ^]^ A memfractive system is mathematically modeled by fractional derivatives and fractional integrals. Such a theoretical model has recently been adopted to explain the highly asymmetric hysteresis *I–V* relationship in ITO/semiconductor/Cu structures including material systems such as lead iodide,^[^ ^80^ ^]^ methylammonium iodobismuthates,^[^ ^81^ ^]^ and tin mixed ligand complex [SnI_4_{(C_6_H_5_)_2_SO}_2_].^[^ 82, 83 ^]^

## 3 Neuron as a Nonlinear Computing Component in Neuromorphic Dynamic Systems

In biology, neuron is a nonlinear processing unit serving as the building block of the nervous systems. About 10^11^ neurons communicate through synapses and cooperate mutually to receive, process, and transmit information, in the way in which our brain forms and becomes the most ingenious system in nature. The realization of artificial neurons is also fundamentally important for implementing artificial neuromorphic systems.

A competent artificial neuron should emulate the critical functions of a biological neuron, namely, integrating information and performing nonlinear transformation. As a result, the neuron in ANN algorithms is usually modeled by a nonlinear activation function after the weighted summation. However, these ANNs are still limited to simple recognition applications and have a much lower energy efficiency compared with the brain. To reduce this gap, enhance the computational complexity, and further improve the energy efficiency of AI systems, an important way is to enrich the dynamics of a single neuron and build more biomimetic neural networks, such as SNNs.

**Figure 4:**
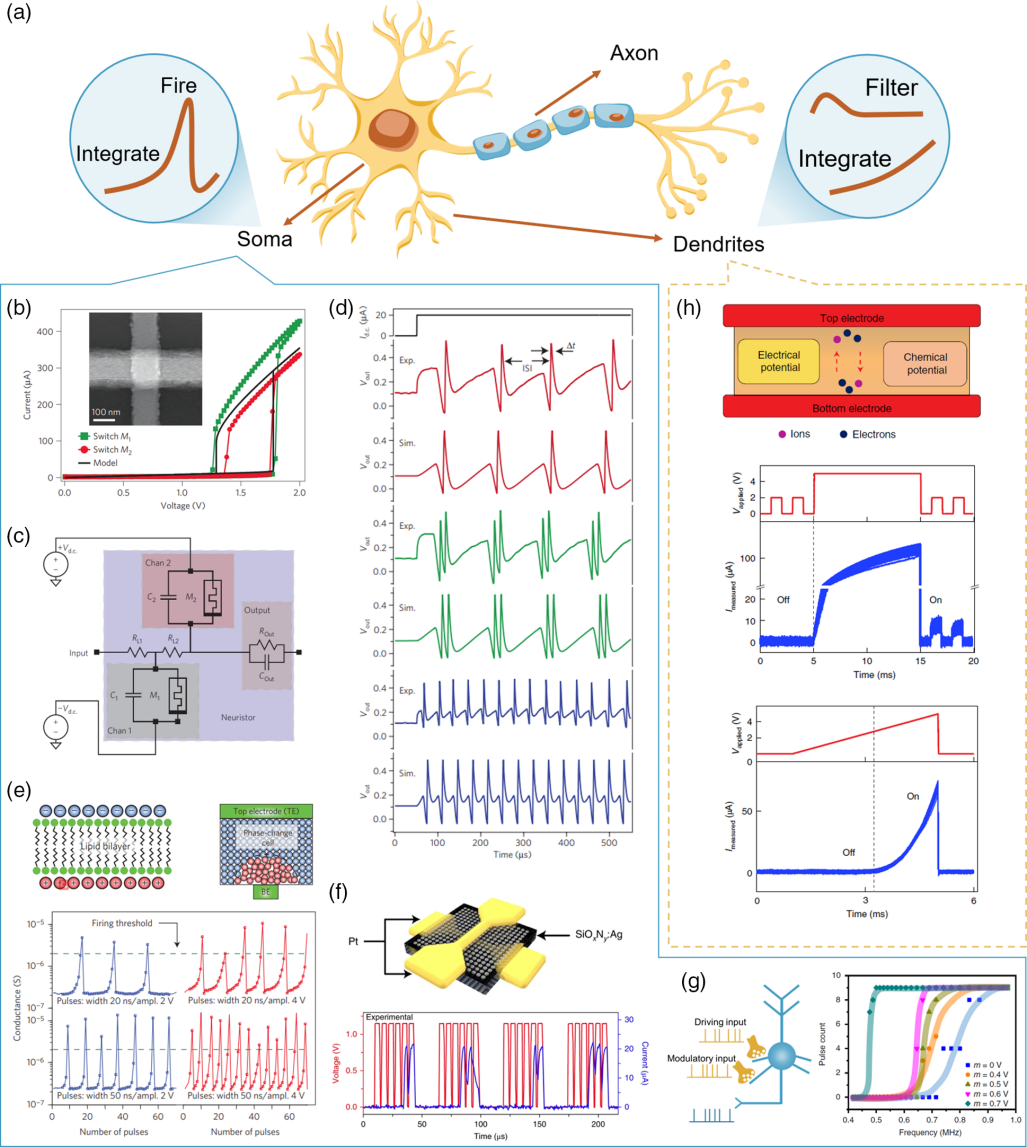
Memristive neuron and dendrite with nonlinear data‐processing property. a) A schematic diagram of biological neuron which is composed of dendrites, a soma, and an axon. b) The thresholding switching character of NbO_*x*_ under voltage sweeping, with a typical scanning electron micrograph in the inset. c) Circuit diagram of the memristive H–H neuron. The channels are emulated by Mott memristors, each is paralleled and are biased with opposite polarity voltage sources. d) Experimental and simulated spike outputs of the memristive H–H neuron with different circuit parameters. e) Response of phase‐change device conductance when a series of crystallizing pulses is applied, which allows the integrate‐and‐fire dynamics in a phase‐change neuron. The phase‐change device is automatically reset to the initial state after its conductance reaches a threshold. f) Illustration of a diffusive memristor and the LIF dynamics of the diffusive memristor artificial neuron. g) Neuronal gain modulation of NbO_*x*_ LIF neuron, showing multiplicative gain modulation when applying different modulatory inputs. h) Schematic illustration of a metal oxide‐based artificial dendrite and its nonlinear current responses to the applied voltage, which show integration and filtering property. b–d) Reproduced with permission.^[^ ^86^ ^]^ Copyright 2012, Springer Nature. e) Reproduced with permission.^[^ ^93^ ^]^ Copyright 2016, Springer Nature. f) Reproduced with permission.^[^ ^9^ ^]^ Copyright 2018, Springer Nature. g) Reproduced under the terms of the CC‐BY 4.0 license.^[^ ^21^ ^]^ Copyright 2020, The Authors, published by Springer Nature. h) Reproduced with permission.^[^ ^116^ ^]^ Copyright 2020, Springer Nature.

A biological neuron is composed of dendrites, a soma, and an axon (**Figure** **4**a).^[^ ^84^ ^]^ Each neuron receives information from the dendrites and then processes and integrates them by updating membrane potential on the soma, and the output signal is transmitted to other neurons through the axon. A neuron carries messages in the form of electrical signals called nerve impulses. When the membrane potential exceeds the threshold, the neuron fires and generates an action potential. This working mode is based on the K^+^ and Na^+^ ions distributed across the membrane. In its resting state, the so‐called ATP pump pumps Na^+^ out of and K^+^ into the cell through the membrane. When every three Na^+^ ions are pumped out, two K^+^ ions are pumped in, resulting in a net negative potential of the membrane in the resting state, which forms the basis of all bioelectricity generation. When the membrane potential reaches the threshold upon integrating external stimuli received through its connected synapses, the voltage‐controlled Na^+^ channels open; hence, Na^+^ ions flow in driven by the concentration gradient built by the ATP pump. The membrane potential is thus depolarized, resulting in the rising edge of the action potential. Subsequently, Na^+^‐ion channels are closed, before which the K^+^‐ion channels start to open, leading to an outflow of K^+^ ions driven by the K^+^ concentration gradient and the formation of the dropping edge of the action potential. At the end of the action potential process described earlier, the neuron returns to its resting state being maintained by the ATP pump.^[^ ^84^ ^]^

### 3.1 H–H Neuron

The H–H model proposed based on the above understanding can well approximate the various spiking behaviors of biological neurons, although the biochemistry behind this picture is even more complicated. H–H model is described by four coupled first‐order differential equations containing four state variables, two of which are gated variables serving as abstraction for Na^+^‐ and K^+^‐ion channels, respectively.^[^ ^85^ ^]^ As shown in Figure 4b,c, these gated elements can be realized by a circuit consisting of two Mott memristors, each of which is in parallel with an external capacitor.^[^ ^86^ ^]^ Such artificial ion channels are coupled through a load resistor and connected to the input resistor and output resistor. Rich biomimetic dynamics can be observed in this artificial neuron, including all‐or‐nothing spiking of an action potential, a bifurcation threshold to a continuous spiking regime, signal gain, and a refractory period (Figure 4d). Totally, up to 23 types of known biological neuron spiking behaviors have been reported in the Mott H–H neuron.^[^ ^87^ ^]^

### 3.2 Leaky‐Integrate‐and‐Fire Neuron

The above H–H neuron based on Mott memristors faithfully mimics the behavior of biological neurons and is more efficient compared with H–H neuron based on CMOS circuits in terms of area and energy.^[^ 86, 87 ^]^ However, the hardware cost can be further reduced in a leaky‐integrate‐and‐fire (LIF) neuron model, which is highly simplified by capturing the basic functionalities of a spiking neuron, while ignoring the specific shape of an action potential. Hardware realization of the LIF model using emerging two terminal devices is attractive due to its high scaling potential and high efficiency.^[^ ^88^ ^]^

The integration in a biological neuron is realized by charging the cell membrane capacitance through ion channels, which is the key to integrating temporal and spatial information. Likewise, a capacitor can be naturally adopted as the electronic counterpart. However, as it is difficult to achieve a high capacitance density in an integrated circuit, an electronic neuron implemented with capacitors may incur a large circuit area. To address this problem, a variety of methods have been proposed utilizing the nonlinear internal physical processes in memristors. Typical implementations of the integration dynamics have been demonstrated based on different types of memristors, such as Mott memristor, redox‐based memristor, ferroelectric memristor, etc. For Mott memristor, the current generated heats the Mott material and induces thermal accumulation when voltage pulse is applied,^[^ 21, 89 ^]^ whereas metal ions can migrate and grow to form a conductive filament that serves as an accumulation process in metal ion‐based redox memristors.^[^ ^9^ ^]^ Instead, the polarization in the ferroelectric layer can also be gradually flipped under voltage stimuli,^[^ 90, 91, 92 ^]^ and the growth of the crystalline phase during the incremental amorphous‐to‐crystalline phase transition induced by electrical pulses acts as the accumulation dynamics in PCM.^[^ ^93^ ^]^ Some other emerging devices have also been adopted to implement artificial neurons. For example, the electrochemical doping process in ion‐gated transistors can be used to emulate the integration dynamics of neurons,^[^ ^94^ ^]^ and the skyrmion racing distance can also serve as the accumulation variable.^[^ ^95^ ^]^

When the potential of the membrane exceeds the threshold, the neuron is expected to fire and release electric pulses. The rising edge of the action potential is triggered by a strong nonlinear transition in memristors, like the abrupt increase in conductance in Mott memristors,^[^ 21, 89 ^]^ diffusive memristors,^[^ ^9^ ^]^ and phase‐change devices,^[^ ^93^ ^]^ when the electrodes are bridged by a conductive channel. It can also be induced by the sudden switch‐on of the FET as a result of sufficient ferroelectric polarization in FeFET.^[^ 90, 91, 92 ^]^ However, the dropping edge of the action potential originates from a volatile inverse process, that is, metal–insulator transition in a Mott memristor^[^ 21, 89 ^]^ or the rupture of Ag filament(s) driven by the minimization of interfacial energy between Ag and dielectrics.^[^ ^9^ ^]^

Finally, implementation of an LIF neuron requires a leaky characteristic. When the external stimulus is not strong enough to directly increase the membrane potential above the threshold value, the membrane potential will leak in the intervals between input signals and gradually decay.^[^ ^90^ ^]^ This leaky feature is enabled by the volatile relaxation processes in the devices, including heat dissipation in Mott devices,^[^ 21, 89 ^]^ ion diffusion in the diffusive devices,^[^ ^9^ ^]^ spontaneous depolarization in ferroelectric layers,^[^ 90, 91, 92 ^]^ as well as backward ion diffusion in the ion‐gated transistors.^[^ ^94^ ^]^ The leaky dynamics in artificial neurons provides a crucial mechanism for spatiotemporal integration of signals at the soma, which contributes to their nonlinear computing functions.^[^ ^96^ ^]^ For instance, the leaky dynamics can differentiate the arrival sequence of different spikes accumulated in the soma, whereas a nonvolatile memory without leaky dynamics typically integrates all spikes equally regardless of their arrival sequence. A large dynamic range is expected to guarantee a pulse output with large amplitude, which in turn facilitates the cascade of neurons and the transmission of information in the system.

It should be noted that not all of the abovementioned devices have the ability to mimic all these stages internally. When a single device cannot fully realize the LIF model, additional assistance is needed. For example, an external reset is required to return the device to the resting state for phase‐change neuron,^[^ ^93^ ^]^ due to its nonvolatile nature. Figure 4e,f shows the spiking behaviors of the neurons based on diffusive memristor and phase‐change memristor, respectively. Other neuronal functions of memristor‐based LIF neurons involving spatiotemporal integration and neuronal gain modulation have also been demonstrated (Figure 4g).^[^ ^21^ ^]^ Artificial neurons implemented by CMOS have been also widely studied, which can be divided into the analog technology route and digital technology route. In an analog CMOS neuron, information integration is usually achieved by a capacitor^[^ ^97^ ^]^ and the nonlinear activation is conducted by an operational amplifier‐based scheme.^[^ 97, 98 ^]^ However, for digital implementation, adders and accumulators are utilized to accumulate the inputs, and a comparator circuit is used to judge the requirement for the spike.^[^ 99, 100 ^]^ It should be noted that both of these techniques suffer from large‐area overhead and high‐energy‐consumption problems compared with the memristive neuron.

### 3.3 Oscillation Neuron

Neural oscillation is a rhythmic or repetitive neuronal activity widely involved in the central nervous system. It can be generated individually or by coupling with other neurons. Neural oscillations play an important role in brain activities, including feature binding, frequency‐encoded information transmission mechanisms, and the generation of rhythm.^[^ 101, 102, 103 ^]^ It is therefore of significance to implement oscillation neurons in hardware in a compact and efficient manner.

To date, oscillation neurons have been demonstrated based on Mott memristors as well, by applying a long voltage bias as the input.^[^ 104, 105, 106 ^]^ Instead, nanoscale magnetic tunnel junctions emit microwave voltages when they are driven by direct current input in a regime of sustained magnetization precession through the effect of spin torque, which also provides a compact nonlinear self‐sustained oscillator that mimics the periodic spiking activity of biological neurons.^[^ ^107^ ^]^ Oscillation neurons can also serve as building blocks in coupled oscillatory networks, as will be discussed below.

### 3.4 Artificial Dendrites

Recent studies have shown that the dendrites in neurons play a non‐negligible role in information processing,^[^ 108, 109, 110 ^]^ besides integration and transmission of input information. This may indicate that the computing power of our brain is hundreds of times stronger than what we expected, due to the majority area of the dendrites being occupied. Incorporating dendritic computing into neuromorphic systems may offer an approach to further enhancing their computation capability. Specifically, the dendrites integrate the spatial and temporal information in a nonlinear manner^[^ 111, 112, 113 ^]^ and meanwhile filter out insignificant background information for better energy efficiency.^[^ 114, 115 ^]^ These functions have been emulated by a two‐terminal Pt/TaO_*x*_/AlO_*δ*_/Al memristor, which can be reversibly switched by modulating the interfacial Schottky barrier induced by the oxygen‐ion drift under electric field.^[^ ^116^ ^]^ As shown in Figure 4h, the electronic dendrite filters out small input voltages in an off‐state, while nonlinearly accumulating the signals after switching on by a large stimulus.

In addition to the behavioral‐level emulation of dendrites including filtering and nonlinear integration, multiterminal neurotransistors have been reported to emulate the structure of the dendritic tree though multiple in‐plane gates.^[^ ^117^ ^]^ The conductivity property of the channel is controlled by these in‐plane gates, which are coupled by strong lateral protonic/electronic capacitive coupling effect through a proton‐conducting solid‐state electrolyte film.^[^ 118, 119 ^]^

With memristive dendrites, sound location^[^ ^119^ ^]^ and recognition task from a natural noisy background^[^ ^116^ ^]^ have been reported. For street view house number (SVHN) dataset, an extra low power consumption of ≈7.84 mW has been reported in neuromorphic computing systems with memristive dendrites, which is 70× lower than the ≈567 mW power consumption achieved by Tianjic chip (CMOS ASIC)^[^ ^120^ ^]^ and more than three orders of magnitude lower than that of the CPU (≈26 W).

## 4 Memristive Reservoir for Nonlinear Feature Mapping

**Figure 5:**
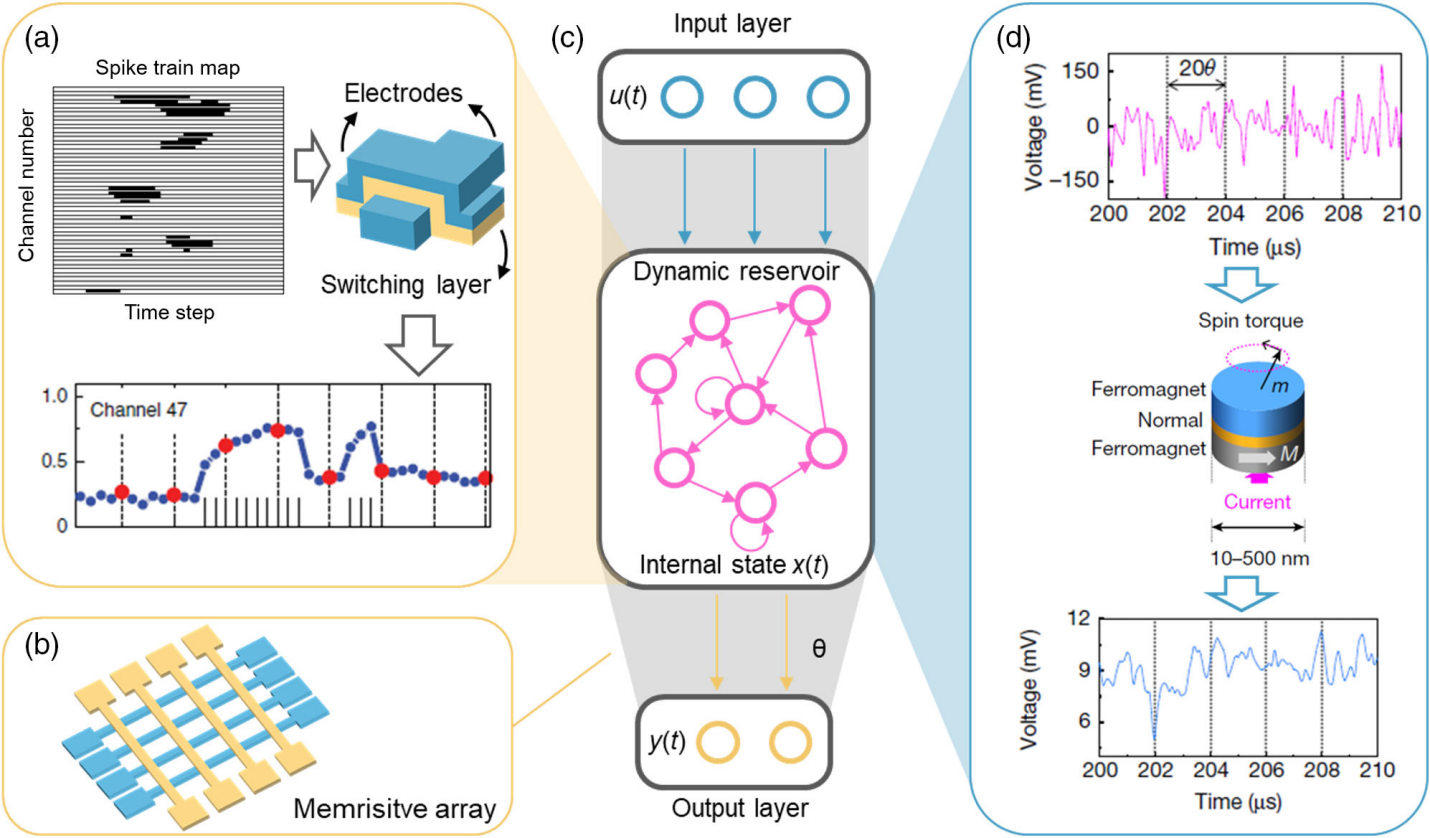
Memristive reservoir for nonlinear feature mapping. a) Temporal response of redox memristors to the spike trains. The information is encoded in the spike trains, which excite the memristor to different conductance states and realize feature mapping of a reservoir. b) Schematic illustration of a memristive crossbar, which can accelerate the linear operation in the readout layer. c) Illustration of a reservoir computing system which consists of reservoir and readout layer. d) Temporal response of spintronic memristors to the spike trains. The information is encoded in the voltage amplitude which excites the memristor to oscillate with different amplitudes and realizes feature mapping of a reservoir. a) Reproduced with permission.^[^ ^130^ ^]^ Copyright 2019, Springer Nature. d) Reproduced with permission.^[^ ^68^ ^]^ Copyright 2017, Springer Nature.

The nonlinearity and dynamics in the above memristors and artificial neurons provide vital substrates for efficient information processing. Generally, neurons in the brain generate transient patterns when excited by input sensory signals. Reservoir computing that mimics this operating mode holds extraordinary advantages in processing temporal information.^[^ 121, 122 ^]^ In general, a reservoir computing system consists of two functional parts, namely, reservoir and readout layer. A reservoir, containing a large number of neuron nodes, is a recurrent network with feedback loops connected by the nodes. These nodes have the ability to process information nonlinearly and have short‐term memory, with which the state of a reservoir is related to the past. As shown in **Figure** **5**c, the input signals *u*(*t*) are randomly connected to the neurons in the reservoir, exciting the reservoir to transient state, which is held by the state of each neuron, denoted as *x*(*t*). Accordingly, the reservoir has nonlinearly projected the inputs to a high‐dimensional feature space (represented by the neuron states). It should be noted that the connection strengths within the reservoir are always fixed, which avoids troublesome training procedures of the recurrent network. Subsequently, these neuron states are read out by a linear weighted sum via the output layer with matrix *θ*. *θ* is the only parameter(s) that needs to be determined by training, which greatly reduces the training workload.

However, there are some limitations to the reservoir. First, the reservoir must have short‐term memory to deal with the temporal sequences. In addition, the reservoir needs to map the different inputs into linearly separable groups in the feature space. However, when the inputs are similar, their projection in the feature should not be too apart; otherwise, it will result in misclassification problems. It means that the reservoir should have good robustness to perturbations. The realization of such reservoirs can be simplified by exploiting the nonlinearity in memristors.

As mentioned earlier, physical implementation of a reservoir necessitates nonlinear responses and short‐term memory. Fortunately, both these two critical features can be easily found in the family of memristors. As shown earlier, a number of redox‐based memristors exhibit nonlinear conductance modulations under identical external pulses and can also have short‐term memory due to spontaneous diffusion of internal ions.^[^ 53, 123, 124, 125, 126, 127 ^]^ In addition, other memristive devices in broad sense including PCM with resistance drift dynamics,^[^ ^128^ ^]^ spin–torque devices with short‐term memory,^[^ ^129^ ^]^ as well as leaky ferroelectric devices^[^ 90, 91 ^]^ can all exhibit a number of states and be utilized as a physical reservoir.

As an example, memristive reservoir based on WO_*x*_ has been adopted to perform a classification task on MNIST dataset and solve a second‐order nonlinear task.^[^ ^130^ ^]^ In this implementation, the input data are transformed into digitalized spike trains, which in turn excite the device to separable conductance states (Figure 5a). Nevertheless, because of the limited conductance states of a single device, a long temporal sequence may not be distinguishable by a final reading after the pulse train, which can thus degrade the overall performance of the classification. Various strategies have been proposed to address this issue, including a segmentation of the long input sequences,^[^ 130, 131 ^]^ utilization of devices with various relaxation constants to extract the correlation in different time scales,^[^ 132, 133 ^]^ and copying the inputs as pulse streams with different frequencies,^[^ ^130^ ^]^ which significantly improved the hardware reservoir. The features mapped into the device conductance can be read out and processed by a linear classifier for the final classification or synthesis. Note that the output layer can also be realized by nonvolatile memristors in a crossbar architecture,^[^ ^131^ ^]^ as shown in Figure 5b.

To further simplify the hardware of the reservoir, the idea of time multiplexing can be introduced. Specifically, the spatially interconnected nodes can be replaced by temporally interacted virtual nodes, which can be enabled by a single nonlinear dynamic node with short‐term memory.^[^ ^122^ ^]^ As the state of a volatile memristor is not only determined by the instantaneous input but also memorized the stimuli in the past, such interactions in time can be equivalent to the connection weights in space, whereas the outputs of a single device at different moments can be regarded as the outputs of different neurons (virtual nodes). As a result, a high‐dimensional reservoir with very low cost of hardware can be constructed by applying time multiplexing to nonlinear devices, which greatly simplifies the circuit.

Based on the time‐multiplexing approach, a single spintronic oscillator has been used for spoken‐digit recognition, with an accuracy comparable with that of state‐of‐the‐art neural networks.^[^ ^68^ ^]^ Herein, the audio waveform is preprocessed and applied to the device as DC current flowing through the junction, as shown in Figure 5d. The resultant oscillating amplitudes with a highly nonlinear relation to input current accompanied by memory effect are detected at different time steps and exploited as the reservoir states. Similarly, this virtual node strategy has also enhanced the ability of WO_*x*_ reservoir on delicate forecasting of chaotic time series.^[^ ^130^ ^]^

The computing capacity of the reservoir can be further enhanced by expanding the width and depth with a hierarchy. In addition, its performance is highly dependent on the device behavior. As a result, the devices for physical reservoir have to possess high endurance and low cycle‐to‐cycle variation. Meanwhile, the nonlinearity of the device should also be intermediate to balance between the effective point‐wise separation ability to different inputs and the robustness to noise,^[^ ^67^ ^]^ and the relaxation time of the device needs to match well with the input frequency to successfully utilize the short‐term memory of the physical reservoir for correlating the events that occur in a specific time scale, which is always important in the implementation of the reservoir hardware. The number of states will determine the sequence length that a single device can distinguish. If the number of states is very limited, the device cannot map different time series to different features which directly correspond to the conductance state of the device. Reservoir computing has also been realized by CMOS analog circuit,^[^ 134, 135 ^]^ FPGAs,^[^ 136, 137 ^]^ and VLSI circuits .^[^ ^138^ ^]^ Compared with CMOS implementations, a memristive reservoir offers much lower energy consumption, and replacing the large number of interconnected neuron nodes by virtual nodes in nanoscale memristors can make the circuit highly compact. The energy efficiency can be further optimized by reducing the programming current of the devices.^[^ ^130^ ^]^

## 5 Coupled Nonlinear Memristive Oscillators

It has been recognized that complex and interesting phenomena can be observed, when a cluster of nonlinear dynamic systems interact with each other. That is actually the situation in our brain: nonlinear neurons are coupled to each other through synaptic connections, based on the fact that there are ≈10^15^ synapses connecting ≈10^11^ neurons in the brain. Instead of going quite complicated and disordered, it is surprising that order spontaneously emerges under certain restrictions. Such order is called synchronization, and the synchronization of neuron clusters has become a very important topic in neuroscience, which can be used to explain important issues, including the mechanism in generating some crucial rhythms, the communication establishment between different brain areas, and so on.

**Figure 6:**
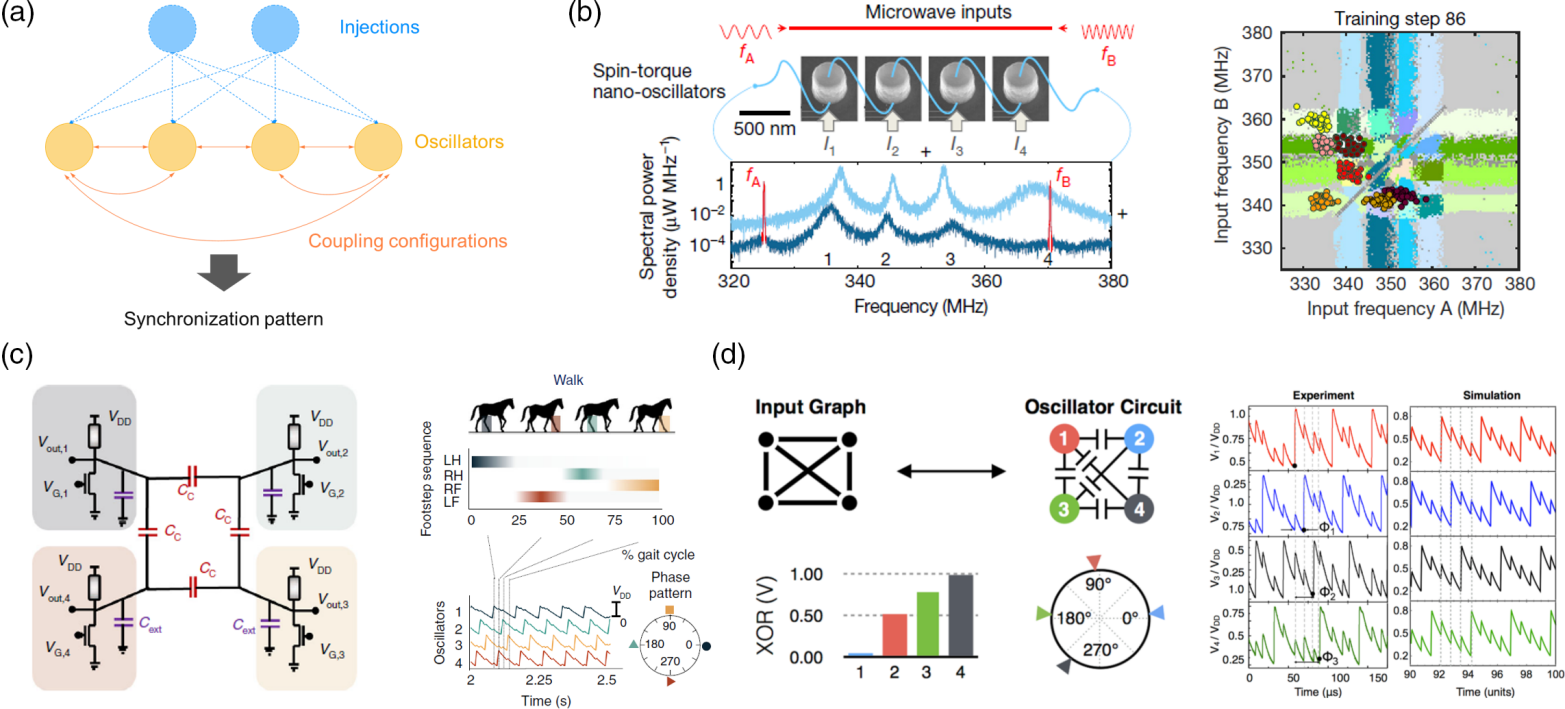
Coupled nonlinear memristive oscillators for computing. a) Illustration of a coupled oscillatory network, whose synchronization pattern is determined by the intrinsic frequency of the oscillators, the coupling configuration, and injected signal. b) A Mott oscillatory network for locomotion control. Four oscillators based on Mott memristors are coupled though capacitances, and the phase order in the synchronization pattern is transformed to footstep sequence. c) A spintronic oscillatory network for vowel recognition. The schematic shows the experimental setup with four spin–torque nano‐oscillators. The oscillators are electrically connected in series and coupled emitted microwave currents. Two microwave signals encoding information in their frequencies are injected to the system through a strip line. d) An memritive oscillatory network and its solution for Max‐cut problem. b) Reproduced with permission.^[^ ^139^ ^]^ Copyright 2018, Springer Nature. c) Reproduced under the terms of the CC‐BY 4.0 license.^[^ ^149^ ^]^ Copyright 2019, The Authors, published by Springer Nature. d) Reproduced under the terms of the CC‐BY 4.0 license.^[^ ^143^ ^]^ Copyright 2017, The Authors, published by Springer Nature.

Synchronization can be described as adjustment of rhythms among different oscillating objects due to their weak interactions. As shown in **Figure** **6**a, these oscillating elements involved must be nonlinear self‐sustained oscillators, which have stable limit cycles in phase space and therefore lead to an immunity to small perturbations. The interaction between these oscillators should be weak enough to maintain their individuality. When the mismatch between the natural frequencies of these oscillators is within a certain range, frequency and phase locking will occur. Utilizing such synchronization dynamics in small networks constructed by coupled oscillators has demonstrated the ability of solving optimization problems and undertaking recognition tasks.^[^ 139, 140, 141, 142, 143 ^]^

As discussed previously, memristors can be used to implement oscillatory neurons, which belong to the category of self‐sustained oscillators. These electronic oscillators can be easily coupled through electrical connections, such as capacitors or resistors. The coupling effect of a capacitor is similar to inhibitory connections between neurons. Therefore, when two oscillators are connected by a capacitor, they tend to oscillate out of phase once the synchronization is achieved. However, resistively coupled oscillators will join the same pace with in‐phase oscillation, once the synchronization is established.^[^ 142, 144 ^]^ Furthermore, spin–torque oscillatory neurons could also be coupled through magnetic field.

A coupled oscillator network implemented by nanoscale memristive oscillators has a low hardware cost, as only one memristive device or a simple memristor‐based circuit is needed to generate oscillations.^[^ ^145^ ^]^ The lateral dimension of the memristor can be scaled down to nanometers. Therefore, building a coupled oscillatory network with memristors is expected to make the oscillatory network system highly compact and energy efficient.

Recent studies in neurobiology have revealed the close resemblance between phase patterns of coupled oscillators and locomotion gaits and discovered central pattern generators (CPGs) based on neuron synchronization.^[^ 146, 147, 148 ^]^ It is found that CPG for the generation of the rhythm patterns for locomotion, breathing, and chewing is located in the spinal cord of vertebrates and in ganglions of invertebrates and contains a lot of neural oscillators. A compact, low‐power CPG is recently demonstrated with VO_2_ devices.^[^ ^149^ ^]^ In this case, the oscillator is realized by a VO_2_ Mott memristor, which is in series with a transistor, forming a 1T1R configuration. The natural frequency of such oscillators can be tuned by applying different voltages on the gate of the transistor. In the memristive CPG, four VO_2_ oscillators are coupled in a ring configuration, where neighboring oscillators are connected by capacitors to achieve interactions. When the voltages applied to transistors are varied, the intrinsic frequencies of individual 1T1R oscillators will change accordingly, which subsequently leads to a changed frequency mismatch among the oscillators and results in different synchronization patterns. As shown in Figure 6b, the output waveforms with inconsistent steps in the beginning gradually evolve into a stable synchronization pattern due to the interaction between the oscillators. These different phase patterns can mimic typical locomotion gait patterns of a horse or a quadruped robot, where the order of the limbs is determined by the phase sequence. The frequency‐tunable VO_2_ oscillator could endow the robot with automatic and real‐time evolution of the control patterns under sensory input signals, allowing an adaptation to the environmental terrain.

These nonlinear oscillatory neurons carry richer dynamics than the highly simplified activation functions in ANN and can therefore endow a smaller network with complex cognitive functions. To conduct a cognitive task, the input, output, and learning strategy has to be designed for coupled oscillatory networks. The input should be involved in the physical dynamics of the network, whereas the output should reflect the collective state of the network after it becomes steady and can be easily read out for the subsequent analysis. More importantly, learning needs to be achieved for cognitive functions. The physical quantity to be learnt must be tunable and memorized according to training and have influences on the dynamic process and the steady state of the network. A potential way is to achieve learning by configuring the natural frequencies of the individual neurons. For spin–torque devices, the DC current flowing through the device can be adjusted to control its oscillation frequency accurately across a wide range.^[^ 68, 139 ^]^ For a Mott oscillator, one can configure its series resistance or adjust the threshold characteristics of the device so as to memorize the knowledge in the oscillation frequency.^[^ ^141^ ^]^ Another way to achieve learning is to store the training information in the coupling strength, which is similar to the synaptic strength between neurons.^[^ ^140^ ^]^ Such learnable coupling strength may be a reconfigurable resistance, which can be easily realized by a nonvolatile memristor.

The input can be external frequency information encoded in electrical or magnetic signals, which are unidirectionally coupled with the network.^[^ 139, 141 ^]^ It can also be represented as temporal information, such as the time delay of the supply voltage/current before initiating the oscillation.^[^ ^140^ ^]^ The output of the network is often provided by the phase information of the steady state, including qualitative synchronization pattern^[^ 139, 141 ^]^ (as shown in Figure 6c) or quantitative phase difference among different oscillators, which can be extracted through the time‐to‐digital conversion module.^[^ ^140^ ^]^ Under this consideration, vowel recognition has been demonstrated using the synchronization of a coupled oscillatory network consisting of four spin–torque oscillators.^[^ ^139^ ^]^ In this demonstration, two formant frequencies of the vowel are encoded in microwave signals with fixed amplitudes and inject into the network with strip line by generating radiofrequency magnetic field that acts on the spin–torque devices. During training, the DC currents dominating individual oscillating behaviors are modified through a supervised learning procedure. The oscillators in the network talk to each other through millimeter‐long wires by microwave currents. The network utilizes the final stable phase pattern as the basis for vowel classification, which is determined not only by the input but also by the network parameters modified through learning. Such vowel classification has also been conducted by replacing the spin–torque devices with VO_2_ oscillators.^[^ ^141^ ^]^ Image recognition task has also been implemented in a resistively coupled VO_2_ coupled oscillatory network based on associative memory, by training the coupling weights with Hebbian learning rule.^[^ ^140^ ^]^ Images are input to the oscillatory network by transforming the pixel grayscale value into time delay of power supply for the oscillator. The memorized pattern can be retraced by the phase information during synchronization. Based on the associative memory ability, these coupled VO_2_ oscillators can be further applied as analog filters in CNNs.^[^ ^140^ ^]^

A coupled oscillatory network gradually converges to a stationary phase or frequency pattern, which shares a similar idea to finding a ground state, analogous to the minimization of the energy function in Hopfield networks.^[^ ^150^ ^]^ This underlying physical process can be exploited to build an Ising machine and solve combinatorial problems. As shown in Figure 6d, an Ising machine based on VO_2_ oscillators has been demonstrated recently.^[^ ^142^ ^]^ The building block of Ising machine, that is, artificial spin, is constructed by a VO_2_ oscillator and a second‐harmonic locking signal injected externally, which guarantees a binary phase state (in‐phase or out‐of‐phase according to the injection). The interaction between spins is mapped to the properties of coupling, where resistive coupling is equivalent to ferromagnetic interaction, whereas capacitive coupling emulates antiferromagnetic interaction. Such a memristive Ising machine has been shown to successfully handle combinatorial optimization problems. Figure 6d shows an example to solve Max‐cut problem by the oscillatory network.^[^ ^143^ ^]^

In addition, the nonlinear mapping relationship between the physical quantities involved in the coupled oscillatory networks can also be used for image processing. A pair‐wise coupled oscillator can efficiently evaluate *L*_k_ fractional distance norm (*k* < 1) for visual saliency processing.^[^ ^151^ ^]^ Coupled oscillatory networks containing more oscillators can perform image‐processing functionalities in high‐dimensional space, for example, color detection and morphological operations such as dilation and erosion.^[^ ^152^ ^]^

Compared with CMOS technology, the memristor‐based oscillatory system holds great advantages in area and energy efficiency, thanks to the potential of implementing ocsillator in a very simple structure. For example, an oscillator can be realized by a single 10 nm spin–torque device, which achieves ultralow power consumption of 1 μW,^[^ ^153^ ^]^ compared with a 10 μm and 8.5 μW CMOS oscillatory neuron.^[^ ^154^ ^]^

## 6 Memristors for Chaotic Computing

Another typical form of dynamics in memristive systems is chaos, which forms the most complex and fascinating part in nonlinear dynamics. Previous studies in neuroscience have indicated that the brain works in the state of “edge of chaos,” which may contribute to the emergence of complexity and other advanced functions of the brain.^[^ ^19^ ^]^ In contrast to the highly abstract neuron models widely used in current ANNs adopting static nonlinear activation functions, biological neurons exhibit various operating modes including phasic and periodic spiking, bursting, self‐sustained oscillations, chaos, and sub/superthreshold active dynamics.^[^ ^107^ ^]^ These rich dynamics, such as ergodicity of chaos, have already been exploited in computing, for example, solving optimization problems.^[^ ^155^ ^]^ The role of these complex dynamics in real intelligence has not been fully unearthed yet; thus, extensive work needs to be done for deeper insights into the human brain. Meanwhile, developing neuromorphic devices with chaotic dynamics and other bioinspired functionalities^[^ ^107^ ^]^ and implementing them for efficient computing are essential for future realization of artificial intelligence that approaches the ultimate efficiency and intelligence of our brain.

**Figure 7:**
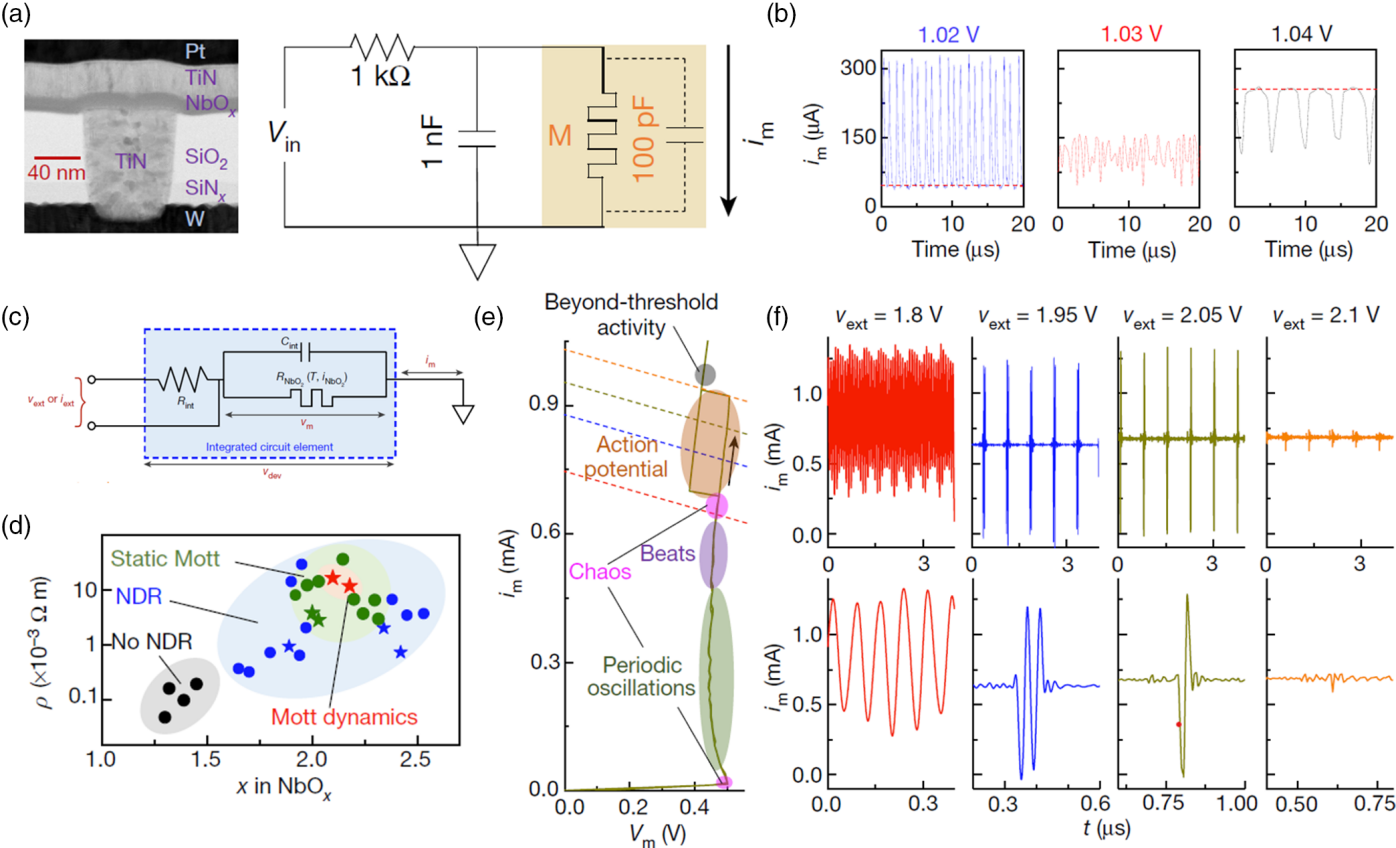
Memristor with local activity. a) Structure of NbO_*x*_ memristor with local activity and schematic illustration of the relaxation oscillator circuit. b) Dynamical behavior the oscillator in (a) with different *V*_in_. The oscillation becomes chaotic when *V*_in_ is 1.03 V. c) Circuit model of the third‐order nanocircuit element. d) Resistivity plotted against the stoichiometry of NbO_*x*_ with corresponding electrical behaviors pointed out. e) Quasistatic current–voltage behavior of third‐order NbO_*x*_ device. f) Measured temporal dynamics of the third‐order NbO_*x*_ device with different applied external voltages. a,b) Reproduced with permission.^[^ ^24^ ^]^ Copyright 2017, Springer Nature. c–f) Reproduced with permission.^[^ ^20^ ^]^ Copyright 2020, Springer Nature.

Mathematically, the generation of chaos in an autonomous continuous‐time system requires three mutually coupled state variables according to the Poincare–Bendixson theorem, which can sometimes also be transformed in the form of a two‐variable system coupled with an oscillator.^[^ 18, 156, 157 ^]^ This can be achieved based on a NbO_*x*_ Mott memristor and a capacitor in parallel, forming a composite device with two variables, namely, internal temperature of the memristor and charge of the capacitor (proportional to the voltage across the device).^[^ ^24^ ^]^ This structure, together with the characteristics of the NbO_*x*_ device, naturally forms a relaxation oscillator, when it is in series with a resistor and powered by a constant voltage supply (**Figure** **7**a). It is found that the thermal fluctuation can serve as an intrinsic oscillating source embedded in this relaxation oscillator and have the ability to induce chaotic behavior. As shown in Figure 7b, when the bias voltage falls within a certain dynamic operation region, chaotic oscillation can be observed (*V*_in_ = 1.03 V). Even richer neuromorphic dynamics can be emulated via a careful adjustment in the structure and material of the device, which has the equivalent circuit model, as shown in Figure 7c. As shown in Figure 7d, when the composition of the material falls within the area marked by the red five‐pointed star, the device can conduct neuromorphic dynamics,^[^ ^20^ ^]^ which is consistent with Chua's theory of local activity.^[^ 156, 157 ^]^ Under this circumstance, the IMT dynamics, that is, the box‐shaped hysteresis in the curve of quasistatic current–voltage of Figure 7e, is further utilized as the third state variable. As shown in Figure 7f, various neuromorphic responses (15 in total) have been observed experimentally in this three‐order nanocircuit element under different voltage biases, according to the parallel load lines in Figure 7e, which are further validated by a compact model.^[^ ^20^ ^]^

As a simple nonlinear circuit element, the memristor has numerous theoretical research in the field of chaotic oscillatory systems.^[^ 158, 159, 160, 161, 162, 163, 164 ^]^ The relevant research based on the mathematical model of memristors provides convenience for quantitatively analyzing the complex behavior of the memristive oscillatory system, such as transient chaotic behavior,^[^ 159, 161 ^]^ synchronization,^[^ ^160^ ^]^ and antisynchronization^[^ ^164^ ^]^ control between memristive chaotic oscillators. It is still a big challenge to experimentally realize these complex chaotic dynamics in devices.

**Figure 8:**
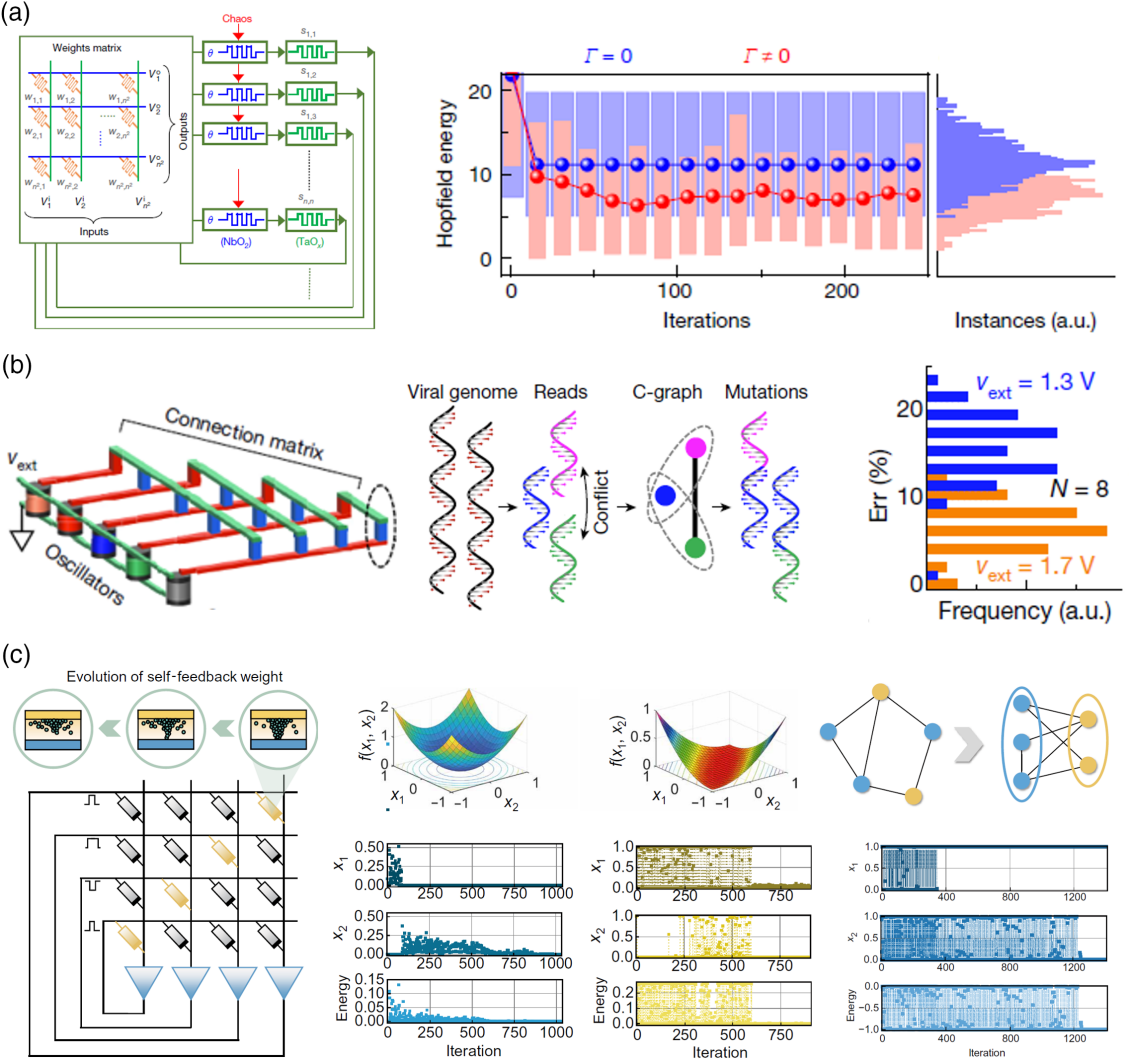
Memristors in chaotic computing. a) Memristive Hopfield network with chaotic neurons and simulation result on traveling salesman problem. b) Memristive oscillatory network with chaotic oscillators for viral quasispecies reconstruction problem. c) Transiently chaotic simulated annealing based on intrinsic nonlinearity of memristors and its experimental results on continuous function optimizations and combinatorial optimization problem (Max‐cut problem). a) Reproduced with permission.^[^ ^24^ ^]^ Copyright 2017, Springer Nature. b) Reproduced with permission.^[^ ^20^ ^]^ Copyright 2020, Springer Nature. c) Reproduced with permission.^[^ ^170^ ^]^ Copyright 2020, American Association for the Advancement of Science.

These dynamic neurons built with nanoscale electronic devices can be assembled into an energy‐based network, for example, Hopfield network, so as to further enhance the computing functions. Hopfield network can solve optimization problems by mapping the task to its energy function. By minimizing the energy function through iterations, the network can automatically find optimal solutions. However, Hopfield network usually faces a common problem of being stuck in local minima. This issue becomes more challenging when the task is complicated and hence has a large number of local minima in the solution space. In this case, replacing the threshold function with a chaotic neuron can introduce fluctuations into the evolution process of the network, therefore endowing the network with ability to jump out of local minima. Simulation results in **Figure** **8**a show that better solutions can be found in the traveling salesman problem by chaos‐aided minimization.^[^ ^24^ ^]^ Similarly, this chaotic oscillatory neuron can also be used as a computing unit in coupled oscillatory networks to help find the ground state when solving optimization problems. Figure 8b shows the improvement after introducing chaos for a viral quasi‐species reconstruction problem in chaotic oscillatory networks.^[^ ^20^ ^]^

It should be noted that there are different approaches to generating such chaotic dynamics in memristive systems. Instead of being generated at the device level and utilized as a local pseudorandom neuron or pseudorandom signal source in computing, the chaotic dynamics can also be generated globally at the network level and adjusted by the bifurcation parameters.^[^ ^155^ ^]^ Again, Hopfield network is taken as an example. Hopfield network consists of a large number of nonlinear neurons coupled via synapses, indicating that a lot of state variables are involved. By introducing proper self‐connections to the neurons, the network will behave chaotically. The dynamic state of the entire network can be tuned by the self‐feedback weights, which serve as the bifurcation parameters. Within a certain range, a strong self‐feedback connection introduces a strong chaos into the network. However, weakening the self‐feedback connection can make the network less chaotic. When the self‐feedback approaches 0, the network degenerates into a classic Hopfield network, which can thus converge with iterations. Overall, the Hopfield network will undergo a transient chaos, if the self‐feedback weight gradually decreases as the network iterates with time. Such transient chaos can be exploited for solution of optimization problems, where the ergodicity of strong chaos in the initial stage is used to fully search the solution space, whereas the self‐feedback weight is weakened afterward to ensure the final convergence of the network. It therefore forms a chaotic simulated annealing (CSA) strategy, similar to the stochastic simulated annealing (SSA),^[^ ^165^ ^]^ by introducing decreasing random noises, which have also been realized based on memristive hardware.^[^ 166, 167, 168, 169 ^]^ However, recent studies have suggested that CSA shows faster convergence speed and gets to lower Hopfield energy compared with SSA.^[^ ^155^ ^]^

CSA can be modeled mathematically as follows.^[^ ^155^ ^]^

(14) $$x_{i}(t)=\frac{1}{1+e^{-y_{i}/\varepsilon }}$$

(15) $$y_{i}(t+1)=ky_{i}(t)+\alpha (\sum_{j=1}^{n}w_{ij}x_{j}(t)+I_{i})-z_{i}(t)(x_{i}(t)-I_{0})$$

(16) $$z_{i}(t+1)=(1-\beta )z_{i}(t)$$

where the three time‐dependent variables $x_{i}$, $y_{i}$, $z_{i}$ are defined as neuron *i*'s output, internal membrane potential, and self‐feedback weight, respectively. $x_{i}$ can be calculated by $y_{i}$ through the sigmoid function with steepness parameter *ε*. Equation (15) describes the iteration of the membrane potential containing three parts. The first part is the leaky item, which means that the history is memorized with a damping factor *k*. The second item represents the input to neuron *i* at time step *t*, including sum of the influence by other neurons $\sum_{j=1}^{n}w_{ij}x_{j}(t)$ and external stimuli $I_{i}$, scaled by a positive parameter *α*. The last item is the newly added self‐feedback aiming to enrich the dynamics of the network, where $I_{0}$ is a positive parameter. Equation (16) offers a strategy to weaken the self‐feedback connection, where the self‐feedback weight is reduced over time, following an exponent relationship with damping parameter *β*.

Through an equivalent mathematical transformation, this algorithm can be well mapped to memristors,^[^ ^170^ ^]^ as shown in Figure 8c. On the one hand, a compact crossbar array is used to accelerate the heavy VMMs involved. On the other hand, the key annealing parameters, namely the self‐feedback connections, are mapped to the devices located in the diagonal positions of the array. These devices are programmed by depression pulses to achieve the cooling schedule of the annealing process. Similarly, it can also be realized with two separate memristive arrays, one of which is dedicated to store the self‐feedback weights in diagonal positions, and the applied voltage can be persistently scaled as weight adjustments.^[^ ^169^ ^]^

As shown in Figure 8c, typical optimization tasks have been demonstrated in this memristive optimizer, including continuous function optimizations on sphere function and Matyas function, as well as combinatorial optimization on Max‐cut problem. It should be pointed out that the cooling curve is critical in the optimization based on simulated annealing approaches,^[^ ^171^ ^]^ which corresponds to the response of device conductance in the diagonal positions during network evolution. As mentioned previously, the conductance response during long‐term depression (LTD) to identical voltage pulses is often nonlinear for a redox‐based memristor, which naturally offers a nonlinear annealing strategy. It is found that the LTD annealing offers a faster convergence and a higher ratio of getting a global optimum, compared with that of linear annealing and exponential strategies. Moreover, the implementation of LTD annealing can be easily achieved by applying simple identical voltage pulses, therefore holding great advantage in hardware implementation.

## 7 Conclusion and Outlook

Here we have reviewed recent studies on utilizing the nonlinearity in memristive devices for the construction of neuromorphic dynamic systems. We discussed the internal mechanisms that endow memristive devices with nonlinearity and rich dynamics and subsequently showed that different neuronal devices and spiking models, including H–H neuron, LIF neuron, oscillation neuron, and artificial dendrite, can be implemented by memristors by exploiting their internal physics and/or chemistry. Such devices form an integral part for building memristive reservoirs, benefitting from the volatility and nonlinearity on the device level, which offers a compact and efficient scheme to process temporal information. Moreover, by coupling memristive oscillatory neurons, the resulting oscillatory neural networks can also be further utilized for CPG and cognitive tasks, etc. Finally, we discussed the complex nonlinear dynamics offered by memristors, including chaos and local activity, which have been used for solving optimization problems efficiently. The discussed dynamic system based on memristors is shown in **Table** **2**.

**Table 2:** A summary of memristor‐based nonlinear dynamic system

Dynamic system	Number of state variables	Device characteristic	Applications
Artificial neuron	≥2	Nonlinear integration; threshold switching; volatile	Spatiotemporal information integration
			Multiplicative gain modulation
Reservoir computing system	≥1	Nonlinear mapping; volatile	Image classification; spoken‐digit recognition; temporal data forecasting
Coupled oscillatory system	≥4	Threshold switching; volatile	Pattern classification; combinatorial optimization; central pattern generation; associative memory; image processing
Chaotic computing system	≥3	Nonlinear chaotic property	Combinatorial optimization

For memristive neurons, some advanced functions such as the refractory period have also been emulated.^[^ ^90^ ^]^ However, the function of the artificial neuron is still not complete compared with a biological one. Lots of efforts are also needed to improve the driving ability of artificial neurons by optimizing the memristor device, which enables the application in multilayer neural network hardware. How complete the function of neuron in an ANN needs to be is yet to be further discussed in the research community.

There are still a few questions to consider before further implementation of memristive reservoirs. First, the training process involved in a reservoir computing system is mainly for the weights in readout layer to reduce the difficulty and cost of training, and it does not mean that the connection relationship within the reservoir is totally static and completely inaccessible by the reservoir designers. On the one hand, in some cases, the random connection within the reservoir cannot achieve optimal performance and the connections have to be designed as a specific structure, including neighboring connections^[^ ^172^ ^]^ or cyclic connections^[^ ^173^ ^]^ However, the current memristor reservoir is contained in a single device. How to achieve these couplings with specific spatial structures through the material structure design and interconnection of the devices requires further consideration by researchers. On the other hand, sometimes it is necessary to pretune the weights inside the reservoir before training the readout layer, such as the spectral radius of the weights.^[^ 174, 175 ^]^ As for memristive computing system, as the dynamic relationship between the input and output of the device was completely determined after the device was fabricated, the influence of this on the scalability and adaptability of different tasks deserves special attention. In addition to the nonlinear mapping function of the reservoir, its own dynamic characteristics should also receive more attention in the physical reservoir computing. Recent study has proven that the reservoir system based on the first‐order‐reduced and controlled‐error (FORCE) learning approach can implement coherent patterns with proper feedback to the reservoir, which exhibits better performance in its chaotic state.^[^ 132, 133, 176 ^]^ Some theoretical research suggests to set the reservoir at the edge of chaos to ensure a neither too expanding nor too contracting mapping between input space and feature space.^[^ 177, 178 ^]^ However, the current memristor reservoir implementations are based on single first‐order memristors without these complex dynamic properties. Using the chaotic memristor^[^ ^24^ ^]^ or high‐order memristor^[^ ^20^ ^]^ to physically realize the reservoir computing system will also be an interesting research topic.

This article has reviewed the rich functions demonstrated by coupled memristive oscillatory systems with a small number of devices. Great advantages in area and energy efficiency have been reported, thanks to the potential of implementing memristive ocsillators in a very simple structure with nanoscale memristive devices. However, the current experimental results are limited to very small systems. How to map a practical scale problem to the oscillatory hardware and realize proper training is the current challenge. Moreover, the method to achieve a proper topology and a high degree of interconnection between the oscillatory neurons is the second challenge faced by the researchers. Spintronic oscillators which could be coupled by various physical quantities including current and magnetic field may offer a possible solution to this issue. In addition, the peripheral circuit to readout and compare the phases also needs elaborate design for system optimization. The current understanding of the role of chaos in the brain is still very limited. To further reveal its mechanism and apply it to the artificial neuromorphic systems require the joint efforts of neuroscientists, computer scientists, and hardware scientists.

Despite the encouraging progress in recent years, this field is still in its infancy. One can see that most of the experiments involved in this Review are still toy demonstrations at the individual device level or very small array level, which are still far from anywhere near the complexity and computing capacity of the brain. There are still a lot of scientific and engineering efforts needed for developing a practical hardware system based on these prototypes. Moreover, it needs to be emphasized that the construction of neuromorphic systems is a highly interdisciplinary task that is closely related to neuroscience advancement in understanding the detailed working mechanisms in the brain. Meanwhile, collective efforts from physicists, material scientists, electrical engineers, and computer science are also critical in further disclosing the device mechanisms and controlling their behaviors on demand. The future development of this field therefore requires close collaborations among different communities and may rely on mutual inspiration between neuroscience research and artificial neuromorphic systems as well. We expect a lot more studies and accomplishments on building neuromorphic systems to come up in the next decade, especially by leveraging the rich nonlinearity and dynamics of memristive devices in general.

## Conflict of Interest

The authors declare no conflict of interest.
